# Factors associated with the choice of public health service among nursing students in Thailand

**DOI:** 10.1186/s12912-017-0202-x

**Published:** 2017-01-23

**Authors:** Krisada Sawaengdee, Nareerut Pudpong, Thunthita Wisaijohn, Rapeepong Suphanchaimat, Weerasak Putthasri, Mylene Lagarde, Duane Blaauw

**Affiliations:** 1 International Health Policy Program (IHPP), Ministry of Public Health, Nonthaburi, 11000 Thailand; 2 Public Health Technical Office, Ministry of Public Health, Nonthaburi, Thailand; 3 London School of Hygiene and Tropical Medicine, London, UK; 4 Centre for Health Policy (CHP), Wits University, Johannesburg, South Africa

**Keywords:** Job choices, Job preference, Job intention, Nursing students, Nursing graduates, Nurses, Public, Private, Rural, Attitude

## Abstract

**Background:**

Despite the fact that public and private nursing schools have contributed significantly to the Thai health system, it is not clear whether and to what extent there was difference in job preferences between types of training institutions.

This study aimed to examine attitudes towards rural practice, intention to work in public service after graduation, and factors affecting workplace selection among nursing students in both public and private institutions.

**Methods:**

A descriptive comparative cross-sectional survey was conducted among 3349 students from 36 nursing schools (26 public and 10 private) during February-March 2012, using a questionnaire to assess the association between training institution characteristics and students’ attitudes, job choices, and intention to work in the public sector upon graduation. Comparisons between school types were done using ANOVA, and Bonferroni-adjusted multiple comparisons tests. Principal component analysis (PCA) was used to construct a composite *rural attitude index (*14 questions). Cronbach’s alpha was used to examine the internal consistency of the scales, and ANOVA was then used to determine the differences. These relationships were further investigated through multiple regression.

**Results:**

A higher proportion of public nursing students (86.4% from the Ministry of Public Health and 74.1% from the Ministry of Education) preferred working in the public sector, compared to 32.4% of students from the private sector (*p* = <0.001). Rural upbringing and entering a nursing education program by local recruitment were positively associated with rural attitude. Students who were trained in public nursing schools were less motivated by financial incentive regarding workplace choices relative to students trained by private institutions.

**Conclusions:**

To increase nursing workforce in the public sector, the following policy options should be promoted: 1) recruiting more students with a rural upbringing, 2) nurturing good attitudes towards working in rural areas through appropriate training at schools, 3) providing government scholarships for private students in exchange for compulsory work in rural areas, and 4) providing a non-financial incentive package (e.g. increased social benefits) in addition to financial incentives for subsequent years of work.

## Background

Nursing shortages are a worldwide phenomenon. The deficiency in availability of nursing professionals is exacerbated by increased demand for health services arising from the epidemiological and demographic transitions as well as the international migration of health personnel [1]. In addition to overall shortages, geographical imbalances contribute to disparities in health outcomes between rural and urban populations [2]. However, the factors affecting these health workforce imbalances are numerous and complex [3]. For economists, the distribution of health professionals is a function of the healthcare labour market. From this point of view, as real wages increase, more health professionals will be willing to be employed and more people will enter the health professions [2]. However, in addition to salaries or living allowance, several studies have suggested that supportive working conditions, such as mentoring/preceptorship, greater autonomy, opportunities for lifelong learning, and rapid career advancement, could enhance health worker retention in rural areas [4–7]. It has also been documented that medical and nursing students born or brought up in rural areas are more likely to choose a job in a rural area [7–11].

Thailand has made remarkable progress in social and economic issues, moving from low-income to upper-middle income status in less than a generation, and it has been one of the widely-cited development success stories, with sustained economic growth and impressive poverty reduction, particularly in the 1980s [12]. During the same period, several human resources for health (HRH) strategies proved successful, particularly in increasing the production of doctors and nurses and their retention in rural areas. However, while the registered nurse (RN) workforce throughout Thailand increased slightly from 1.5% in 2008 to 1.76% in 2014 [13], there are several pressures on the nursing workforce [14]. First, the geographical mal-distribution of nurses remains a problem, especially in the more remote hardship and border areas. To promote rural retention of RNs, in addition to normal admission modes (e.g. national entrance, direct admission, local demand, and special talent), local recruitment as suggested by WHO [15] was adopted to attract local high school students to enter nursing schools and then return home after graduation.

Second, many RNs are approaching retirement age. The latest data from the Thailand Nursing and Midwifery Council (TNMC) show that 25% of RNs were over the age of 54, which is close to a typical RN retirement age. Also, the workload for RNs in public health service is usually high, and that contributes to burnout and early departure from the nursing profession. As many younger nurses leave their career, this results in a wider generation gap (between new graduates and senior nurses) and an aging workforce in the public sector [14]. In response to these pressures, Thailand will need 60,000 additional RNs by 2020 to meet the demand. Therefore, the TNMC plans to increase annual nurse production from 7600 (in 2012) to 10,000-12,000 enrolments during the period of 2015 to 2020 [14].

In 2014, there were 86 nursing schools in Thailand; 23 are private and 63 are public [13]. Public nursing schools contribute 75% of the production capacity and fall under three different ministries: 30 schools are under the Ministry of Public Health (MOPH), which mainly produce nurses for provincial and rural health services throughout the country; 29 are under the Ministry of Education (MOE) that produce nurses to serve the university hospitals; and five are under the Ministry of Defence (MOD) that produce nurses for their own institutions. The private provision of healthcare has expanded with the introduction of policies promoting Thailand as a medical hub [14]. Consequently, private nursing schools, that mostly serve the domestic private health sector, have increased their role in producing nurses in response to the medical hub policy [16]. There has been a substantial increase in private nursing schools. In 2006, 19.6% of new nurses graduated from private nursing schools, and this increased to 24.1% in 2010 [17]. This shows the growing importance of the private sector’s contribution to nurse production in Thailand [14, 18].

Thailand’s health system is a mix of public and private health services. Nevertheless, the majority of health service facilities are public (approximately 80%). Private facilities are mostly concentrated in the capital city and large cities in urban areas, serving patients with a greater ability to pay who do not want to wait in long queues. Ninety-five percent of public facilities are owned by the Ministry of Public Health (MOPH), most of which are located in rural areas. At the same time the demand for private health services is growing rapidly, and there are increased incentives to attract nurses to work in the private sector [19]. According to the private pay survey in 2013, for a new RN, private hospitals offer a basic monthly salary which is approximately 5000 baht (approximately 140 USD) higher than public hospitals [20]. This does not include additional financial incentives, which vary from hospital to hospital (e.g. bonus, additional allowances for specialized care, and career advancement opportunities), which are normally less available in public hospitals. The “pull factors” in the private sector have had negative consequences for the recruitment and retention of RNs in the public sector, particularly in rural areas where they are needed most. Pull factors have also produced institutional imbalances for the public sector as some public facilities (e.g. big regional and university hospitals) have more nursing staff because of higher prestige, better working conditions, more ability to generate additional income, or other situation-specific factors, while the smaller district hospitals are understaffed [3]. This phenomenon has occurred in Thailand; as private health facilities keep expanding [16], public training institutions alone may not be able to produce a sufficient number of qualified nurses to address the demand in the public sector. This has also led to the increasing role of private training institutions to increase the supply of nurses for both the private and public sectors.

The integration of new nurse graduates into the workforce is crucial to address nursing shortages and maintain the delivery of public health services. Therefore, understanding the job intentions and choices of new entrants to the nursing profession, and the factors influencing those decisions, is important in policy formulation aimed at ensuring an adequate supply of nurses to meet population healthcare needs. That understanding will also help nurse educators to structure appropriate learning experiences and help public health sector leaders to allocate resources needed to improve the recruitment of adequate numbers of nurses to meet the needs of communities.

Therefore, this study sought to: (1) examine Thai nursing students’ attitudes toward rural practice and their intention to work in public service after graduation, (2) determine factors affecting their choices and intentions to work, and (3) assess whether and to what extent there are differences in the students’ attitudes and job choices between the public and private training institutions.

## Methods

A descriptive, comparative, cross-sectional sample survey was used to study the job intentions of nursing students from public and private nursing training institutions. The data were collected between February and March 2012.

### Sampling

This study was conducted in a sample of 40 nursing schools out of the total of 60 existing MOPH, MOE and private nursing schools in Thailand. We excluded 13 new nursing schools which had not yet graduated students and five public schools established to produce nurses for hospitals under the MOD and Bangkok Metropolitan area (Table 1). Nursing schools were selected randomly, stratified by geographical area and type of school (Public MOPH, MOE and private).

**Table 1:** Characteristics of the sampling frame and sample of the nursing institutions

	Region	MOE		MOPH		Private		Total	
		Total	Sample	Total	Sample	Total	Sample	Total	Sample
Number of Institutions	South	3	2	5	3	0	0	8	5
	Central	3	3	9	5	6	4	18	12
	North-East	3	2	6	5	4	3	13	9
	North	3	2	7	4	2	3	12	8
	Bangkok	3	2	2	1	4	3	9	6
	Total	15	11	29	18	15	11	60	40
Number of students	Total	3270	2337	2560	1365	2210	1257	8743	4954

In each school sampled, all nursing students were invited to participate in the study. The study sample was composed of 4954 final-year students, representing 56.7% of all final-year nursing students in Thailand in 2012.

### Survey instruments

Two types of data collection instruments were used in this study: an institutional assessment questionnaire completed by the administrator of each training institution, and a self-administered questionnaire for all nursing students in the selected schools who agreed to take part in the survey. Both questionnaires were sent to the targeted nurse training institutions and the focal point (an officer who worked in that institution and was well-informed about the study process) of our study team took responsibility for distributing the questionnaires to the study samples, collecting them, and then sending them back to the study team. The survey instruments were developed following discussions with experts and educators in nursing and public health higher education in Thailand.

The institutional assessment tool included questions on the source of funding, the school’s performance as assessed by its pass rate (proportion of students who passed the national licensing examination), staffing levels, and staff qualifications.

The student survey was divided into two parts. The first part included questions on the socio-demographic characteristics of respondents: age, gender, domicile during childhood, type of school attended and its location, type of entry into the nursing school,…etc. The second part included questions on their attitudes towards working in rural areas, their job intentions immediately after graduation and withinthe next five years, and the reasons for their job choices after graduation.

### Analysis methods

Descriptive statistics were used to summarise the characteristics of both institutions and nursing students, using proportions for categorical variables, and means or medians for numerical data. We applied the Chi-square test, analysis of variance (ANOVA), and the Kruskal-Wallis test, as appropriate, to evaluate differences in these variables between the three types of training institutions: MOPH, MOE, and private sector.

In terms of the key study outcomes, we evaluated students’ job intentions immediately after graduation and within the next five years by calculating the proportion selecting each option, and then used the Chi-square test to compare students from the three types of institutions. Secondly, we examined respondents’ top three reasons for choosing a job by creating a ranking score from their responses -- scoring 3 points for first choice, 2 for second, 1 for third, and 0 for other-- so that higher ranking scores indicate more important reasons. The mean ranking of scores for different reasons were compared between the three groups using ANOVA, and Bonferroni-adjusted multiple comparisons tests to identify which differences were statistically significant. Lastly, we investigated students’ attitudes towards rural areas. We calculated the proportion who ‘Agreed’ and ‘Mostly Agreed’ with various positive and negative statements about rural areas. We also used principal component analysis (PCA) to construct a composite *rural attitude index* combining 14 of these questions. Cronbach’s alpha was used to examine the internal consistency of the scales. To improve the alpha, three less-related items (no. 3, 5, and 7) were removed, and the final test scale of the remaining 11 items generated an alpha of 0.8. The differences between the mean scores across students from the three institution types were assessed using ANOVA.

These relationships were further investigated through multiple regression. A logistic regression model was developed to identify factors predicting students’ choice of a public job (immediately and within 5 years after graduation), while linear regression models were used to investigate predictors associated with students’ ranking of income as a motivation in their job choices, and the PCA-derived *rural attitude index*. In addition to the type of training institution, other characteristics selected for the models included admission by local recruitment or not (direct admission, local demand, and special talent combined), sex, age, rural domicile during childhood, students’ motivation by money (for job choice and rural attitude models) and financial burden during their studies (having or not). Results are reported as odds ratios for the logistic models and as coefficients for the linear regression. For all analyses, a *p*-value less than 5% was regarded as significant. All data analysis was performed using STATA software version 11.

## Results

### Institutional profile

Out of the 40 schools targeted by the survey, 36 agreed to participate in the study, giving a response rate of 90% overall. Of the four refusing to participate in the study, three were from MOPH and one was private.

The top panel of Table 2 presents the characteristics of these institutions. Out of the 36 nursing schools surveyed, 41.7% (*n* = 15) were under MOPH, 30.6% (*n* = 11) were under MOE and 27.8% (*n* = 10) were private nursing schools. Most schools were located outside of Bangkok. Only public nursing schools, MOPH and MOE, had admission policies setting specific quotas for students originating from rural areas. Both types of public institutions were heavily subsidized by the government, with a median subsidy of 66.8% (MOPH) and 61.3% (MOE). There were four types of admission to nursing education in this study: (1) “National entrance” refers to students who undertook the central national entrance examination to get a seat in a nursing education institution, (2) “Direct admission” refers to students who applied directly to the university located close to their hometown, (3) “Local demand” refers to students who were admitted based on a request from a local authority, such as the provincial health office, and (4) “special talent” refers to students who had a special talent, such as an outstanding athlete or musician. Thus, “rural recruitment” in this study was ‘direct admission’, ‘local demand’, and ‘special talent’ combined. The results also show that MOE institutions include the highest proportion of teaching staff with a PhD, at 37.1%.

**Table 2:** Characteristics of institutions and nursing students in three different type of school

Characteristics	MOPH	MOE	Private	All groups	*p*-value^a^
Institutions					
Observations	15	11	10	36	
School location					
% Outside BKK	93.3	72.7	60.0	22.2	0.129
% BKK & Vicinity	6.7	27.3	40.0	77.8	
% Schools with admission policy focusing on geographical difficulties	93.3	45.5	0.0	52.8	<0.001
% staff with PhD degree	9.4	37.1	13.0	18.1	<0.001
Median % of budget funded by government	66.8	61.3	0.0	63.0	0.751
Median pass rate (final exam) in 2011	96.7	86.4	64.0	88.9	0.010
Median pass rate (1^st^national licensing exam) in 2011	49.0	59.8	24.2	45.5	0.003
Nursing students					
Observations	1308	1139	902	3349	
Mean age	22.9	22.9	23.4	23.0	<0.001
% Female	93.9	95.4	95.2	94.8	0.209
% living in rural area during childhood	54.3	45.1	48.6	46.6	<0.001
% Admission					
by National entrance	66.3	32.6	29.3	44.9	<0.001
by Direct admission	26.0	49.3	66.4	44.8	
by Local demand	6.9	13.3	1.0	7.5	
by Special talent	0.8	4.9	3.3	2.9	
% Funding source					
Government scholarship	16.3	7.3	10.4	11.7	<0.001
NGO scholarship	22.4	14.9	2.5	14.5	
Self & Family Support	47.9	53.0	50.3	50.3	
Any loans	13.4	24.7	36.9	23.5	
% School location					
Outside BKK	80.6	63.5	50.4	66.7	<0.001
BKK & vicinity	19.4	36.5	49.6	33.4	
Expenditure on tuition fees (Median in Thai Baht^b^)	200,000	200,000	500,000	250,000	<0.001
% reporting a financial burden	30.6	27.1	28.1	28.7	0.141

^a^Statistical significance of results across groups tested using ANOVA for means, Kruskal-Wallis for medians, and the chi-squared test for proportions

^b^approximately, 30 Thai baht = USD 1

*MOPH* Ministry of Public Health, *MOE* Ministry of Education

To compare institutions in terms of the quality of outputs, we evaluated the median pass rate for the final examination, set individually by each nursing training institution, and for the national licensing examinations for each group. For the institutions’ final examination, the highest pass rates were achieved by MOPH institutions (nearly 97%), followed by MOE (86%) and then private institutions (64%). Students who successfully pass their institution’s final exam are then allowed to sit for the national licensing exam. The national licensing exam is organized three times a year, and the result of the first exam is generally used as the best measure of quality of an institution’s output. Results from Table 2 show that graduates from private institutions do quite poorly, with only a quarter successfully licensed after the first exam, while the median pass rate amongst MOPH graduates is nearly 50% and that amongst MOE graduates is the highest at nearly 60%. These differences were statistically significant. Interestingly, it seems that less than half of nursing graduates were licensed to work as nurses, at least immediately after graduation.

### Student profile

Of all 4954 students from the surveyed institutions, 3349 students completed the survey questionnaire, amounting to a total response rate of 68.5% (respectively, 77.1%, 62.2% and 67.4% amongst MOPH, MOE and private students).

Respondents were predominantly female (94.8%) and young, with a mean age of 23 years. About half of students in all school types had lived in a rural area during their childhood, but this proportion varied from 45.1% in MOE schools to 54.3% in MOPH schools. Finally, looking at modes of admissions into the nursing schools, two thirds of nursing students in MOPH schools were admitted through national entrance, against only a third in the other two types of institutions. By contrast, the majority of nursing students in MOPH and private schools were recruited directly by the institutions.

### Job intentions

**Fig. 1:**
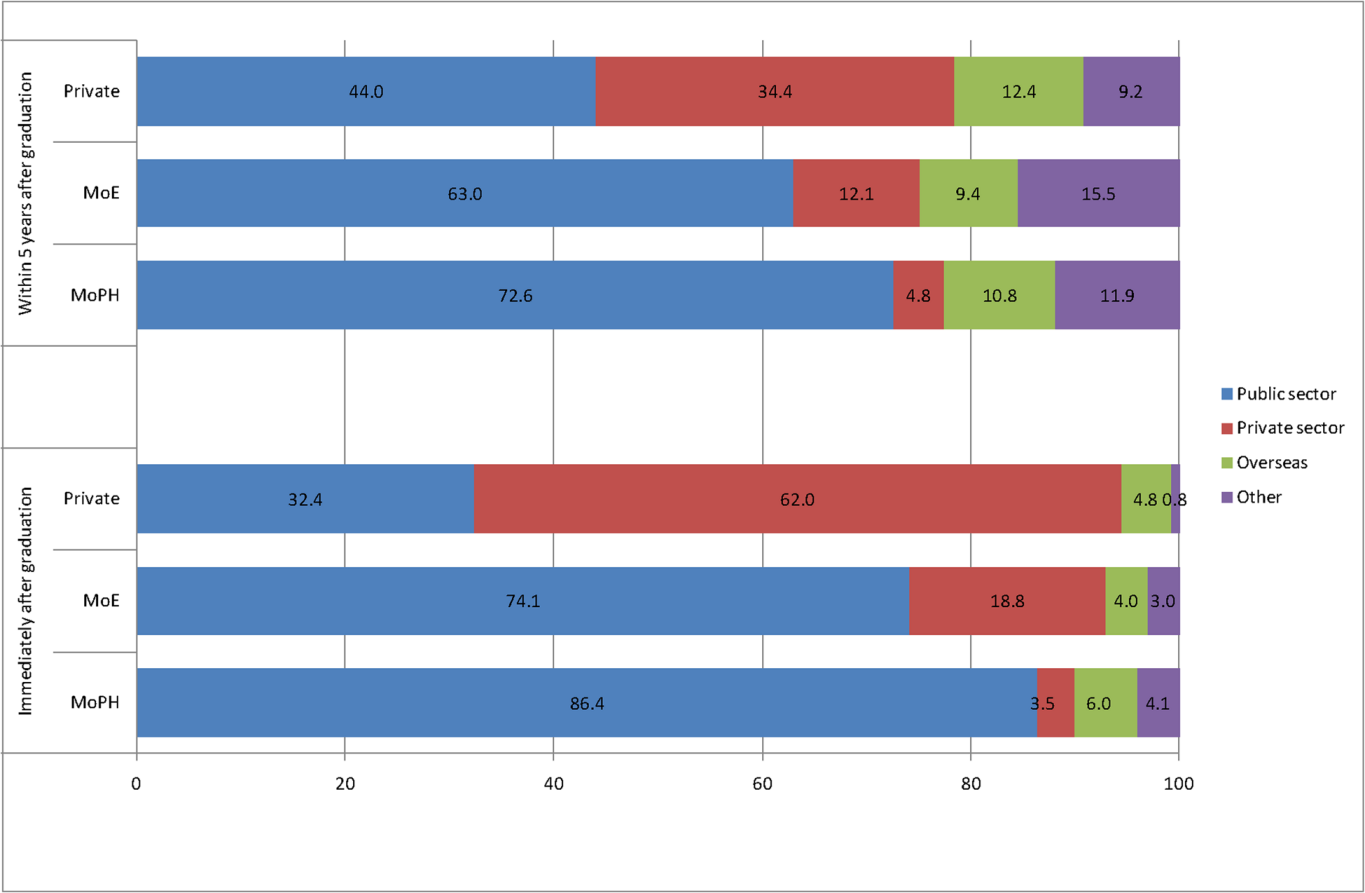
Job intentions immediately after graduation and within 5 years after graduation

Figure 1 presents the reported job intentions of students immediately after graduation (below) and within the next five years (above). Immediately after graduation, a large proportion of MOPH and MOE students, respectively 86.4% and 74.1%, reported that they would prefer to practice nursing in the public sector. By contrast, 62% of private students declared they would want to practice in the private sector. Less than 10% of all students reported that they wished to go abroad or leave the nursing career.

When asked about their job intentions within the five-year period following their graduation, a lower proportion of students from MOPH and MOE schools said they intended to practice nursing in the public services (respectively 72.6% and 63%), but a larger proportion of privately-trained students (44%) reported they would be interested in a job in the public sector within five years after graduation.

### Reasons for job choices

Table 3 shows the average ranking score of each of the suggested reasons for choosing a job after graduation. The findings show that “a good income” was ranked the highest overall, earning an average score of 0.969. Interestingly, students from private nursing schools appeared significantly more motivated by the idea of a good income, compared to students from MOPH schools (*p* < 0.001) and MOE schools (*p* < 0.05). Having “good advancement opportunities” was the second most highly rated reason for all students and, similarly, we find that privately-trained students gave more importance to that aspect than those who trained in public training institutions (*p* < 0.001 for both comparisons).

**Table 3:** Ranking scores for top three reasons for selecting a job

Reasons	Average ranking score [Mean (SD)]^a^				*p*-value (ANOVA)			
	MOPH	MOE	Private	All Groups	Overall	Multiple Comparisons		
						MOPH-MOE	MOPH-Priv	MOE-Priv
Good income	0.878 (1.069)	0.961 (1.028)	1.111 (0.961)	0.969 (1.031)	<0.001	0.133	<0.001	0.003
Good advancement opportunities	0.855 (1.135)	0.926 (1.130)	1.156 (1.154)	0.960 (1.145)	<0.001	0.364	<0.001	<0.001
Good job-related benefits	0.645 (1.058)	0.843 (1.148)	0.857 (1.189)	0.769 (1.129)	<0.001	<0.001	<0.001	1.000
Good opportunity for continuing education	0.741 (1.130)	0.788 (1.127)	0.860 (1.200)	0.789 (1.149)	0.056	0.947	0.049	0.865
Proximity to friends/family	0.755 (1.058)	0.594 (0.994)	0.542 (0.963)	0.643 (1.016)	<0.001	<0.001	<0.001	0.764
Social responsibility/duty	0.744 (1.143)	0.520 (1.024)	0.326 (0.834)	0.555 (1.040)	<0.001	<0.001	<0.001	<0.001
Chance to return to childhood domicile	0.708 (0.972)	0.433 (0.877)	0.343 (0.792)	0.516 (0.908)	<0.001	<0.001	<0.001	0.071
Good environment	0.290 (0.835)	0.320 (0.853)	0.381 (0.915)	0.325 (0.864)	0.048	1.000	0.043	0.325
Attractive living environment	0.202 (0.675)	0.377 (0.897)	0.405 (0.897)	0.316 (0.822)	<0.001	<0.001	<0.001	1.000
Independent/remoteness from family	0.044 (0.335)	0.048 (0.356)	0.080 (0.455)	0.055 (0.378)	0.067	1.000	0.080	0.183

^a^A ranking score of respondents’ top three reasons for choosing a job were created by scoring 3 points for the first choice, 2 for the second, 1 for third, and 0 otherwise, and so the high ranking scores indicate more important reasons

### Attitudes towards working in a rural area

Table 4 reports the attitudes of nursing students for various aspects of working life in rural areas. Most students agreed with statements indicating a positive attitude towards working in a rural area. Overall, nursing students across all groups agreed that despite the lack of social and leisure amenities (item 11), and the limited support in one’s job (item 2), rural communities were characterized by having very friendly people (item 6) and working there would not limit one’s ability to communicate with one’s peers. A larger proportion of students from MOE schools agreed with the idea that their school had prepared them well to work in rural areas (item 9) and had inspired them to work in rural hospitals (item 10).

**Table 4:** Proportion of respondents who agree and mostly agree with the statements

Statements		MOPH	MOE	Private	*p*-value^a^
1	Working in rural areas provides opportunities to use various skills	61.9	67.2	65.9	0.016
2	There are supportive environments when working in rural areas	30.5	30.7	30.0	0.945
3	Working in rural areas limits communications with professional peers	38.7	36.5	35.0	0.198
4	Working in these areas provides opportunities to work independently	60.5	67.9	56.9	<0.001
5	There is a lack of amenities and entertainment in rural areas	44.2	49.5	46.6	0.035
6	People in rural areas are friendly	95.0	96.1	95.1	0.346
7	Working in rural areas results in “isolation” from friend and family	35.6	37.8	32.7	0.061
8	Working as a nurse in hospitals in rural areas is the most important contribution to health of population	83.8	90.8	87.5	<0.001
9	Nursing school prepared me well to work in rural areas	75.6	66.8	68.1	<0.001
10	Nursing education inspires me to work in hospitals in rural areas	50.9	43.6	41.8	<0.001
11	There are abundant amenities and entertainment in rural areas	88.1	86.3	82.3	0.890
12	Working in hospitals in rural areas is most challenging	70.5	76.5	71.4	0.002
13	Working in hospital in rural areas provide opportunities for real-life problem solving	71.4	78.5	72.8	<0.001
14	Overall, you have a positive attitude towards working in rural areas	74.9	81.0	78.6	<0.002

^a^Statistical significance of results across groups using the chi-squared test

### Factors associated with the choice of public jobs

Table 5 shows the results of multivariate analysis looking at the factors associated with intention to work in the public sector immediately after graduation (Model 1), within the next five years (Model 2), preference for money as a reason for choosing a job (Model 3), and positive attitude towards rural areas from the PCA index (Model 4).

**Table 5:** Regression analysis of factors predicting job preference of Thai nursing students

	(Model 1)		(Model 2)		(Model 3)		(Model 4)	
	Intend to work in the public sector immediately after graduation		Intend to work in the public sector 5 years after graduation		Average score for “good income” as a reason for choosing a job		Composite index for attitude towards working in rural areas	
	OR (95% CI)	*p*-value	OR (95% CI)	*p*-value	Coeff (SE)	*p* value	Coeff (SE)	*p* value
MOPH (vs Private)	13.184 (10.454−16.628)	<0.001	3.393 (2.786−4.132)	<0.001	−0.258 (0.048)	<0.001	−0.105 (0.085)	0.217
MOE (vs Private)	6.015 (4.899−7.385)	<0.001	2.199 (1.823−2.654)	<0.001	−0.159 (0.047)	0.001	−0.083 (0.084)	0.324
Local recruitment (vs National entrance)	0.996 (0.831−1.194)	0.968	1.083 (0.925−1.268)	0.322	−0.059 (0.038)	0.124	0.188 (0.068)	0.005
Rural domicile during childhood (vs Not)	1.218 (1.025−1.448)	0.025	1.317 (1.134−1.530)	<0.001	−0.123 (0.036)	0.001	0.171 (0.064)	0.008
Female (vs Male)	2.056 (1.422−2.972)	<0.001	1.818 (1.311−2.522)	<0.001	−0.132 (0.082)	0.109	0.465 (0.145)	0.001
Aged over 25y old (vs < 25y)	1.398 (0.818−1.391)	0.221	0.873 (0.528−1.443)	0.597	−0.211 (0.127)	0.099	−0.293 (0.224)	0.191
Motivated by money ranking score	0.723 (0.666−0.745)	<0.001	0.933 (0.868−1.002)	0.057			−0.134 (0.031)	<0.001
Had financial burden (vs Not)	0.823 (0.681−0.995)	0.044	1.010 (0.856−1.192)	0.907	−0.062 (0.040)	0.126	0.087 (0.071)	0.222
Constant								
Observations	3150		3156		3219		3183	
Pseudo R-squared	0.190		0.048		0.015		0.018	
*p*-value	<0.001		<0.001		<0.001		<0.001	

Results of the two models looking at factors associated with intention to work in the public sector (Models 1 and 2) confirm that graduates from public nursing schools are much more likely to report wanting to work in the public sector, either immediately after graduation or within the next five years. Specifically, the odds of wanting to work in the public sector immediately after graduation are 13 times higher when a nursing student graduates from a MOPH school, and six times higher when they graduate from a MOE school, compared to a private sector student. The differences between public and private nursing schools become less marked for job intentions within five years after graduation, with MOPH and MOE graduates having only 3.4 and 2.2 times, respectively, the odds of wishing to work in a public sector facility compared to private graduates.

The Model 1 results also show that being a woman and having spent one’s childhood in a rural area was associated with a greater likelihood of wanting to work for the public sector, either immediately after graduation or in five years. By contrast, the importance of financial concerns, as captured by preference for a good income or having a financial burden, is associated with a lower likelihood of wanting a public sector job.

Turning to factors associated with ranking good income as more important in choosing a job (Model 3), the multiple regression results confirm that nursing students from public institutions have a much lower preference for this reason for a job, compared to private students. We found that being from a MOPH school reduced the ranking score associated with “a good income” as a reason for choosing a job by nearly 0.258 points, while being from a MOE school reduced it by 0.159 points. Similarly, students who grew up in rural areas seemed to care less about a good salary, compared to students who grew up in an urban area. Other characteristics were not found to be statistically significant.

Finally, looking at Model 4, the results show that there was no difference between nursing students who trained in a public or a private institution, with regard to their attitude toward working in a rural area. On the other hand, there was significant evidence that a rural upbringing, being a woman and having being recruited locally by the nursing school were all associated with more a positive attitude towards rural areas. Lastly, being financially motivated was significantly associated with a less positive attitude towards rural areas.

## Discussion

There are five main findings of this study in regard to the final year students’ career choice for taking a nursing job after graduation. Firstly, having trained in a familiar environment was shown to be an enabling factor for subsequent job choices among Thai nursing students. Those trained in public nursing schools were more likely to choose to work in the public sector, either immediately, or within the next five years after graduation. In Thailand, generally, public nursing students have training experience in only public hospitals, while private students have experience in both public and private hospitals during their studies. Students may intend to work in a familiar environment. This finding may be explained by preconceived expectations influencing job preference [21]. Focusing on the student-to-nurse transition period, new graduates may feel insecure and inadequately prepared to meet the demands of the nursing role [22]. Therefore, they are likely to choose working in the familiar places that they experienced while studying. Moreover, nursing students in this study intended to work in Thailand rather than going abroad, with less than 10% of all students reporting an intention to go abroad or leave the nursing profession. This finding is in contrast with those found in other Asian and African countries [8, 23]. Approximately 28% of medical and nursing students in Bangladesh, Ethiopia, India, Kenya, Malawi, Nepal, the United Republic of Tanzania and Zambia expected to migrate abroad [8]. Another study in Uganda also found that 70% of nursing students would like to work abroad (e.g. the US or UK) for better financial remuneration [23]. In addition to working in a familiar environment in the home country, the reluctance to go abroad may be partially explained by the relatively weaker English language skills among Thai nursing students. Previous literature indicated that Thai health personnel, including nurses, still needed to improve their English skills to gain confidence in working with foreign patients [24, 25]. Hence, “nurse brain drain” may not be a major problem in Thailand at present.

Secondly, a positive attitude towards rural working in a rural area was a strong predictor of intention to work for the public sector among nursing students in all school types, and this tendency probably builds throughout their training. This finding is consistent with the theory-based model of Ajzen and Fishbein, which contends that intention to work in particular area is generally influenced by attitudes and subjective norms [26]. Thus, nursing education has the potential to influence student attitudes toward working in a rural area. For example, a study in Canada showed that the use of a photovoice approach (where students are required to take photographs that represent challenges and facilitators of rural nursing practice, and then engage in written reflection about their photos) for a rural nursing course was proven to be useful in fostering students’ exposure to, interest in, and understanding of rural settings and their influence on rural nursing practice [27]. Provision of comprehensive rural education during training has the potential to encourage nursing students to be more appreciative, informed, prepared, and interested in selecting and remaining in rural nursing positions post-graduation.

Thirdly, this study confirmed that rural recruitment of nursing students is a significant strategy for attracting and retaining students to serve in rural areas. Rural upbringing and entering the nursing education program by local recruitment were found to be associated with a more positive attitude toward rural areas in this study, and that finding is similar to studies with newly graduated doctors in Thailand and in other low-middle income countries (LMICs) [8, 9]. These studies found that students admitted to medical school through a special quota for rural background students preferred to work in community hospitals in rural areas, and students who have spent a significant period of time in rural settings were more likely to practice in their home country and in rural areas. Similarly, a study in South Africa found that rural-born nurses were more likely to choose a job in rural areas [7]. This finding is also supported by the survey conducted in more than 300 colleges with a nurse practitioner training program in the USA, which emphasized rural recruitment and practice as key to addressing rural health workforce shortages, and so encouraged both public and private sources to support this [10]. A systematic review of factors that influence a career choice in primary care among medical students from high-, middle-, and low-income countries also found that previous exposure to a rural area and having a rural background are among the common factors that facilitated their decision to work in a rural area [11]. In addition, this study reinforced the WHO recommendation that rural recruitment is an effective HRH strategy to address rural retention [15].

Fourthly, financial support is important for education and likely to influence a nursing student’s job choice, particularly those trained in private schools. Hence, government subsidy and/or scholarship for private education and bonding services may help attract and retain nurses in public service after graduation. In this study, it is no surprise that “good income” was the most common reason for choosing a job as money is important for everyone. However, the study found that private students were more concerned about income and intended to choose jobs in the private sector, especially immediately after graduation. Students who were trained by the MOPH and MOE were less likely to choose their job based on salary. In part, this difference may be because students in private schools incur significantly higher expenses and debt than students in MOE and MOPH institutions. Indeed, almost 40% of students in private nursing schools reported relying on education loans. Thus, receiving a scholarship from the government for their nursing education, and bonding services (as a condition of the scholarship) may help attract and retain private nurse students to work for the public sector.

Finally, to promote rural retention, financial incentives are important, but not the only mechanism. A combination of financial incentives with other strategies has proved to be more successful. In this study, the opportunity for career advancement was the second most common reason for job selection among all participants. This finding is consistent with several previous studies which found that provision of other supportive working conditions is crucial for nurse retention [4–7]. For example, having mentoring/preceptorship (beyond standard orientation) is very important to help student adjustment during the beginning of their nursing education [4]. In addition, lifelong learning opportunities, more autonomy, and faster career advancement are among the non-financial incentives that can help retain nurses in the profession [5–7]. Hence, the combination of both financial and non-financial incentives should be provided for nurse students and graduates to maximize retention and help fill labor shortages in that sector, and especially in rural areas.

## Conclusions and policy implications

A shortage of nurses has the potential to severely impede the future improvement of the health of the Thai population. Despite a marked success in boosting the production of nurses in the past, the nurse shortage and maldistribution of nurses still remains a key public health concern in Thailand, especially in the context of a rapidly aging population. This study found that to tackle such a problem, some key policy options are worth exploring, and these are not limited to just the public sector. Indeed, the private sector can play an important role to help address this problem. Due to the fact that the Thai health system is largely publicly-funded and given the high demand for nurses in public outlets, especially in rural areas, several policy options based on the findings of this study are as follows: 1) nursing schools should continue to selectively recruit students from rural areas as they are more likely to choose to work for public institutions; 2) curriculum content and training experience in nursing schools should be more focused on promoting a positive student attitude toward rural areas by providing more exposure to rural practice and environments during their education; 3) the government could provide scholarships to private students with a period of compulsory work in rural areas after graduation; and 4) since salary appears to be the main determinant of job choice for nursing students, particularly right after graduation, there needs to be a greater focus on non-financial incentives (e.g., mentoring, in-service training, awards, advancement potential) to attract nurse graduates and retain them in the public sector. Public healthcare providers may not be able to compete with private hospitals on wages, but the additional recruitment/retention strategies of tuition reimbursement and earning more social benefits of public service more quickly might prove significant [28].

### Study strengths and limitations

The large number of participants in the survey is a key strength for this study. The response rate was high enough to provide valid description and analysis. However, not all possible variables that influence career choice were included in this survey. Also, variables such as job intentions, attitude towards working in a rural area and reason for choosing a job after graduation, were assessed from students’ self-reporting of their perceptions. A positive response bias in favoring social expectations and in keeping with the Thai culture, may have contributed to some over-reporting of positive attitudes and intentions.

More research is needed to examine the reasons behind students’ career choices, pattern of nurse mobility between sectors, and the most effective measures to recruit new graduates into the public health services workforce. Views from other stakeholders, such as policy makers, faculty, as well as the users of nurses (including patients and professional colleagues at the facilities) should be included in future studies. A longitudinal study to follow up these graduates after entering the health workforce is needed to provide more empirical evidence of the linkage between job intention and actual job selection.

## Abbreviations

ANOVA: Analysis of variance
HRH: Human Resources for Health
MOD: Ministry of Defence
MOE: Ministry of Education
MOPH: Ministry of Public Health
PCA: Principal component analysis
RN: Registered nurse
TNMC: Thailand Nursing and Midwifery Council
